# Towards the Construction of a Mathematically Rigorous Framework for the Modelling of Evolutionary Fitness

**DOI:** 10.1007/s11538-019-00602-3

**Published:** 2019-04-04

**Authors:** Oleg Kuzenkov, Andrew Morozov

**Affiliations:** 1 Department of Mathematics, University of Leicester, Leicester, UK; 2 Shirshov Institute of Oceanology, Moscow, Russia; 3 Lobachevsky State University of Nizhni Novgorod, Nizhniy Novgorod, Russia

**Keywords:** Evolutionary fitness, Selection, Inherited strategy, Measure, Age structuring

## Abstract

Modelling of natural selection in self-replicating systems has been heavily influenced by the concept of fitness which was inspired by Darwin’s original idea of the survival of the fittest. However, so far the concept of fitness in evolutionary modelling is still somewhat vague, intuitive and often subjective. Unfortunately, as a result of this, using different definitions of fitness can lead to conflicting evolutionary outcomes. Here we formalise the definition of evolutionary fitness to describe the selection of strategies in deterministic self-replicating systems for generic modelling settings which involve an arbitrary function space of inherited strategies. Our mathematically rigorous definition of fitness is closely related to the underlying population dynamic equations which govern the selection processes. More precisely, fitness is defined based on the concept of the ranking of competing strategies which compares the long-term dynamics of measures of sets of inherited units in the space of strategies. We also formulate the variational principle of modelling selection which states that in a self-replicating system with inheritance, selection will eventually maximise evolutionary fitness. We demonstrate how expressions for evolutionary fitness can be derived for a class of models with age structuring including systems with delay, which has previously been considered as a challenge.

## Introduction

The concept of evolutionary fitness is one of the best-known paradigms in theoretical approaches to the description of biological evolution and adaptation (Birch 2016; Gavrilets 2004). Charles Darwin himself stated the famous principle of the ‘survival of the fittest’, indicating the key role of selection of life traits or behavioural strategies against certain criteria which can be formalised mathematically via some fitness function (Darwin 1859). The quantitative description of evolutionary fitness came first from the seminal works of Wright (1932, 1988), in which biological evolution was visualised as a ‘hill-climbing’ process in a certain parametric landscape, through which natural selection in the population increases its fitness (Birch 2016; Davies et al. 2012; Gavrilets 2004; Roff 1992). Usually, fitness is defined as the expected number of offspring produced by an individual that will survive until reproductive age (Mangel and Clark 1988; McNamara et al. 2001). Eventually, the process of evolution under these settings should lead the population to evolve to the nearest local maximum of the fitness landscape, and then stop. For this reason, the concept of fitness is central to evolutionary modelling: given a particular fitness function, the evolutionary outcome can be predicted using an optimisation procedure to find the life traits or behaviours which maximise it.

However, the choice of fitness for a subpopulation is generally subjective and strongly depends on the personal preference of the modeller. For example, one can claim that to find optimal life traits, we should maximise (taking into account some possible constraints) the individual reproductive value (Fiksen and Carlotti 1998; Mangel and Clark 1988; McNamara et al. 2001), the growth rate (or minimise the mortality rate) (Han and Straskraba 1998, 2001), the ratio between the food intake and mortality (De Robertis 2002; Gilliam and Fraser 1987; Sainmont et al. 2015) or a certain entropy function (Levich 2000). It probably comes as no surprise that the use of different optimisation criteria can potentially produce conflicting outcomes (Kuzenkov and Ryabova 2015; Morozov and Kuzenkov 2016). In addition, more advanced game-theoretical approaches in evolutionary modelling—which take into account the strategies of the competitors—may suffer from the same drawback since the resultant evolutionarily stable strategy (ESS) generally depends on the choice of fitness—as defined by the pay-off matrix (Broom and Rychtar 2013; Hofbauer and Sigmund 1998). Another shortcoming of the idea of optimising a certain fixed criterion such as the individual reproductive value is that such an approach does not take into account the possible impact of the evolution of species traits on the biotic and abiotic components of the environment (McNamara et al. 2001). For example, a successful behavioural strategy can result in the temporary proliferation of a population, but this may cause a corresponding increase of the population of their predators which can cause a decline in the original population (Morozov and Kuzenkov 2016; Sandhu et al. 2017).

Because of the influence of environmental feedbacks on evolution, there is a general understanding in the literature that modelling the evolution of life traits or behaviour should be somehow linked to the underlying population dynamics equations. For example, the evolution of frequencies of competing strategies can be modelled using a replicator equation (Karev 2010). The individual-based modelling approach explicitly considers the reproduction, spatial movement, competition and mortality of each individual within a population or group (Hellweger et al. 2016): this approach, however, is usually computationally demanding and does not allow us to obtain analytically tractable solutions (Hamblin 2013). A popular approach which links population dynamics with evolutionary processes and allows analytical treatment of the problem is adaptive dynamics. Adaptive dynamics is based on the concept of invasion fitness, defined as the long-term per capita invasion rate of an initially rare mutant strain introduced into an environment determined by a resident strain (Geritz et al. 1997, 1998; Morozov and Adamson 2011; Parvinen et al. 2006). The relative simplicity of the adaptive dynamics framework comes at a price: the method is only applicable to an ergodic environment and assumes rare mutations which make small steps in trait space (Metz et al. 1992). Given these assumptions, and assuming that the overall amount of mutants in the system is small, pairwise comparisons between a resident and a mutant population can be made. However, these simplifications are not always biologically justified (Waxman and Gavrilets 2005): for example, often natural selection occurs via simultaneous competition among large numbers of strains with comparable population densities.

A generic approach to modelling selection in self-reproducing systems should allow us to deal with arbitrary types of life traits (either scalar or function-valued) across a different set of initial conditions and external forcing, in the case where the environment is not necessarily ergodic, for example. One promising approach to modelling natural selection considers dynamics of the measures of sets in the underlying space of traits (Gorban 2007). The outcome of evolutionary modelling is described by the long-term dynamics of measures of sets of inherited elements, rather than the comparison of a number of ‘pure’ life traits against each other. Note that in the case of the presence of a large number of strategies, one can approximate them by a continuum framework.

In this paper, we extend the original ideas of Gorban (2007) and revisit the concept of evolutionary fitness in self-replicating systems in the case where the space of inherited units is an arbitrary function space (encompassing scalar life trait parameters, as well as function-valued behavioural traits and their combinations). We first introduce the ranking order of competing sets of strategies using the underlying population dynamic equations. Then we propose a mathematically rigorous definition of evolutionary fitness, which is based on the ranking order of strategies. We show that the connection between measures and densities in the space of strategies presents a number of mathematical challenges which should be taken into account when modelling selection processes using the density-based approach. Using the new formulation of evolutionary fitness, we formulate the variational principle of modelling selection, which postulates that the long-term outcome of a selection process will correspond to the strategy or trait which maximises evolutionary fitness. Establishing variational principles in this way when modelling biological evolution has a long history with various approaches proposed (Crow 1981; Levich 2000; McNamara et al. 2001; Metz et al. 1992, 2008; Stankova et al. 2013; Wilhelm et al. 1994), but unlike the situation in mechanics, or optics, the framework for developing a unifying variational principle for modelling selection is still missing. Finally, with the help of a few insightful examples, we show how evolutionary fitness can be derived for a class of models with age structuring including delay, which has previously been considered as a mathematical challenge.

The paper is organised as follows. In Sect. 2, we introduce the generic framework of modelling natural selection in systems with inheritance. In particular, we introduce a mathematically rigorous definition of evolutionary fitness. In Sect. 3, we demonstrate how this evolutionary fitness can be found for a class of population models with age structure. In Sect. 4, we discuss the advantages of the proposed concept of fitness when modelling evolutionary dynamics and compare this concept with those from earlier approaches.

## Defining Evolutionary Fitness

### Setting the Stage: The Space of Inherited Units and Measures

Here we introduce a generic modelling concept as well as some important assumptions necessary for a mathematically rigorous definition of evolutionary fitness in a self-replicating system with inheritance.

#### Assumption 1

We consider a self-replicating system, where any set of inherited units (strategies) *v* belong to a compact metric (or compact Hausdorff topological) space *V*.

Biologically, inherited units can be genotypes, behavioural strategies, functional traits, etc. Mathematically, an element *v* can be a scalar, a vector, a function or a vector of functions.

In the case where the number of inherited units *v* is large (or infinitely large or even uncountable), it is impossible to follow the dynamics of each element separately. In other words, for an arbitrary model, it will be meaningless to compare some finite number of inherited units against each other and such a comparison will not inform us about selection in the system (Gorban 2007). Instead, we must consider the evolution of some subsets *A* of space *V*.

#### Assumption 2

We assume that the system $\Sigma$ consisting of any subsets *A* from the space *V* form a $\sigma$-algebra known as the Borel algebra.

To accurately describe the evolution of an inherited unit (or a set of such units), it is necessary to be able to quantify its presence at any moment of time. In the simplest case, we can quantify the presence of strategies via the total population size or total biomass of all strategies $v\in A$. However, we can also use the logarithmic scale of the biomass, or alternatively, we can characterise the presence of strategies in the population via any positive power of population size which can be strategy dependent. In this case, it is rather natural to use the mathematical concept of measure. More, accurately:

#### Assumption 3

Let us assume that at each moment of time *t* each set *A* in $\Sigma$ can be described by a nonnegative function $\mu (t)(A)$. We postulate that this quantity should satisfy the following requirements:

- A value of zero at any given moment of time indicates the absence of *A* in the population.
- A positive value indicates the presence of the set *A* in the population.
- It is countably additive.
- It is a smooth function of time.
- If it tends to zero, this signifies extinction of *v*.

Under the above assumptions, the function $\mu (t)$ should be understood as a Borel measure defined on *V*.

#### Assumption 4

We assume that $\mu (t)$ is uniformly bounded by a constant, i.e. $\mu (t)(V) \le c$ for any *t*.

This is a natural assumption to make, taking into account resource limitations for the population. Using the above assumptions, we can define a selection process.

#### Definition 1

(*Selection*) We have selection of some set of $A\in \Sigma$ in the space of inherited units in the case where the measure $\mu (t)$ of the complement of *A* tends to zero for large times whereas the measure of *A* does not, i.e.

1 $$\begin{aligned} \lim _{t\rightarrow \infty } \mu (t)(V/A)=0 \quad \text {and} \quad \lim _{t\rightarrow \infty } \mu (t)(A)\ne 0. \end{aligned}$$

#### Remark

According to the above definition, the measure of the whole set *V* does not tend to zero: this would signify the extinction of the whole population, which we want to avoid.

#### Remark

The above limit for $\mu (t)(A)$ may not always exist in some systems, for example, in the case of a periodical evolutionary succession of strategies.

Using the measure dynamics, we can compare selective advantages of different sets from $\Sigma$.

#### Definition 2

(*Ranking order of sets*) We state that set $A\in \Sigma$ is better (fitter) than set $B\in \Sigma$ ($A\succ B$), if

$$\begin{aligned} \lim _{t\rightarrow \infty } \frac{\mu (t)(B)}{\mu (t)(A)}= 0. \end{aligned}$$

The introduced relation satisfies the axiom of transitivity, since if ($A\succ B$) and ($B\succ D$), then

$$\begin{aligned}&\lim _{t\rightarrow \infty } \frac{\mu (t)(B)}{\mu (t)(A)}= 0;\\&\lim _{t\rightarrow \infty } \frac{\mu (t)(D)}{\mu (t)(B)}= 0; \end{aligned}$$

and

$$\begin{aligned} \lim _{t\rightarrow \infty } \frac{\mu (t)(D)}{\mu (t)(A)}= \lim _{t\rightarrow \infty } \frac{\mu (t)(D)}{\mu (t)(B)}\frac{\mu (t)(A)}{\mu (t)(B)}= 0; \end{aligned}$$

therefore ($A\succ D$). Moreover, it is impossible that ($A\succ B$) and ($B\succ A$). Therefore, the relation is a partial ranking order in $\Sigma$, not a full order because we cannot compare two sets if the limit of the ratio of their measures is not equal to zero. In the case of the bounded measure ($\mu [t](V)<c$), this definition signifies that *B* will be eventually replaced by *A* because

$$\begin{aligned} \lim _{t\rightarrow \infty } \mu (t)(B)= \lim _{t\rightarrow \infty } \mu (t)(A)\frac{\mu (t)(B)}{\mu (t)(A)}\le \lim _{t\rightarrow \infty } c\frac{\mu (t)(B)}{\mu (t)(A)}= 0; \end{aligned}$$

and there is the selection process of *V* / *B*.

The introduced ranking order of sets may depend on initial conditions. This can be seen from the following illustrative example.

#### Example 1

Consider a system of three competing species $V=(v_1, v_2, v_3)$ with normalised numbers of individuals $\mu _i$ (i.e. the total number of the population is equal to unity $\mu _{1}+\mu _{2}+\mu _{3}=1$). The model equations are of the replicator type

$$\begin{aligned} \frac{\mathrm{d} \mu _i}{\mathrm{d}t}=\mu _i \mu _{i+1} -\mu _i(\mu _1\mu _2+\mu _2\mu _3 +\mu _3\mu _1), i=1,2,3. \end{aligned}$$

where $$\mu _4 \equiv \mu _1$$.

Let the measure of the presence of every element be the normalised number of individuals. In the case $\mu _1(0)=0$ the system becomes

$$\begin{aligned} \frac{\mathrm{d} \mu _2}{\mathrm{d}t}= & {} \mu _2^2 \mu _3, \\ \frac{\mathrm{d} \mu _3}{\mathrm{d}t}= & {} -\mu _2^{2}\mu _3. \end{aligned}$$

For large times we have $\mu _2 \rightarrow 1$, $\mu _3 \rightarrow 0$, hence $v_2\succ v_3$. We can similarly prove that in the case where $\mu _2(0)=0$ we have $v_3\succ v_1$ and in the case where $\mu _3(0)=0$ we have $v_1\succ v_2$. In other words, the ranking order depends on initial conditions. Moreover, when all species are initially present, they can coexist at the equilibrium (1 / 3, 1 / 3, 1 / 3), i.e. no ranking order can be established between the three strategies. On the other hand, in the case of the inverse time ($t \rightarrow -t$), the internal equilibrium loses its stability and the trajectory gradually approaches the boundary of the standard triangular simplex. In this case, all species persist in the system. More examples on dependence of the ranking order on initial condition can be found in Kuzenkov and Ryabova (2015).

Note that the introduced ranking order is not a perfect comparison because a part of *A* may be worse than *B*. To cover this situation, we will introduce the following definition of strong ranking.

#### Definition 3

(*Strong ranking order of sets*) Set *A* is strongly better than set *B* if *A* is better than *B* and any $\mu$-nonzero subset of *A* is better than *B*.

In this case, any nontrivial part of *A* is better than *B*. The strong ranking satisfies the properties of transitivity and anti-symmetry, i.e. we introduce a partial order in $\Sigma$. This gives us the opportunity to compare elements of different sets.

#### Definition 4

(*Ranking order of strategies*) Let any neighbourhood of elements *v* and *w* be $\mu$-nonzero. Element *v* is better than element *w* ($v\succ w$) if there exist neighbourhoods of these elements (*O*(*v*) of *v* and *O*(*w*) of *w*) such that *O*(*v*) is strongly better than *O*(*w*).

It is obvious that this relation also satisfies the properties of transitivity and anti-symmetry, i.e. it introduces the partial ranking order in *V*. The ranking order of strategies may also depend on initial conditions.

If the measure is uniformly bounded ($\mu (t)(V)<c$) and $v\succ w$, it follows from Definition 4 that the measure $\mu$ of some neighbourhood *O*(*w*) of *w* vanishes with $t \rightarrow +\infty$.

Using the above ranking order definition, we can now postulate evolutionary fitness as follows.

#### Definition 5

(*Evolutionary fitness*) In the case where there exists a functional *J*(*v*) which preserves the ranking order of strategies it is referred to as *evolutionary fitness*, i.e. from $J(v)>J(w)$ it should follow that $v\succ w$.

#### Remark

One can easily see that the above definition of fitness is not unique: any function of *J* would be considered as an evolutionary fitness if it preserves the ranking of strategies. It is easy to see that in this case, any strategy $v^*$ maximising the fitness will be the same.

#### Remark

A generic definition of evolutionary fitness would depend on what we determine as the measure of the presence.

Only strategies $v^*$ having the highest ranking order according to Definition 4 will remain after a long time, with the others eventually vanishing. Let *A* be a set from $\Sigma$, such that the closure of *A* does not contain any point $v^{*}$ of the global maximum of *J*(*v*). Then $\mu (t)(A) \rightarrow 0$ and there is selection of *V* / *A*. We emphasise that this selection does not depend on the choice of measure on *V*, provided that assumption 3 is satisfied.

Maximisation of evolutionary fitness *J*(*v*) provides the variational principle of modelling evolution dynamics: among all elements *v*, only the strategies $v^*$ realising the global maximum of *J*(*v*) will survive in the population over time. Thus, finding the evolutionary optimal strategies will be equivalent to finding the maximum value of *J* across *V*.

### Measure Dynamics and Measure Densities

Under the above assumptions, the set of different measures $\mu$ over *V* is a Banach space, where the norm of $\mu$ is equal to its total variation. Therefore, we can now consider the following equation of the dynamics of measure $\mu (t)$

2 $$\begin{aligned} \frac{\mathrm{d}\mu (t)}{\mathrm{d}t} =F(\mu ,t), \end{aligned}$$

where *F* is an operator describing the rate of change of $\mu (t)$ in time.

However, the description based on (2) is too generic to allow us to obtain mathematically rigorous but meaningful results; in particular, the equation does not impose any restriction on mutation rates. In this paper, we will investigate the particular scenario, where inheritance is strong so that we can initially neglect the effect of mutations and assume that offspring have the same genotype as their parents.

An example of a system with strong inheritance is the following generic equation considered in Gorban (2007)

3 $$\begin{aligned} \frac{\mathrm{d}\mu (t)}{\mathrm{d}t}=K(v,\mu ,t)\mu (t), \end{aligned}$$

where $K(v,\mu ,t)$ is a continuous function of $v\in V$ which represents the reproduction coefficient. From (3) one can conclude that if the measure of the set of strategies *A* is initially zero, it will always remain zero, i.e. new strategies cannot be produced by the existing strategies.

However, model (3) cannot describe another important case of strong inheritance, which allows for the effects of delays. For example, the current absence of a particular strategy *v* at some developmental stage/age within the population does not necessarily signify that such a strategy will not appear later due to the maturation of younger individuals with the same genotype. A generic class of models of self-replicating systems with delay and strong inheritance is given by

4 $$\begin{aligned} \frac{\mathrm{d}\mu (t)}{\mathrm{d}t}=\sum _{i=1}^{n}a_i(v,\mu ,t)\mu (t-\tau _{i}^{*})+K(v,\mu ,t)\mu (t), \end{aligned}$$

where the first term provides the rate of replenishment of the distribution due to the maturation of offspring; the delays $\tau _{i}^{*}$ describe maturation time lags or effects of modification of the environment in the past. We assume that we know the history of the measure dynamics $\mu (t)$ in the time interval $[-T,0]$, where $T=\max (\tau _{i}^{*})$. Equation (4) is an extension of (3) describing the possibility of inheritance with a zero measure at the initial moment of time ($t=0$).

Mathematically, it is generally difficult to explore the dynamics of measures directly from Eqs. (3) or (4). However, often we can model measure dynamics using the density distribution across the space of inherited units. To be able to use the density-based modelling framework, we need to assume the following

#### Assumption 5

Let the measure $\mu (t)$ be absolutely continuous with respect to some fixed measure $\mu ^{*}$. In this case, there is an integrable function $\eta (v,t)$ (called the density of the measure) such that

$$\begin{aligned} \mu (t)(A)=\int _{A}\eta (v,t)\mu ^{*}(\mathrm{d}v), \forall A\in \Sigma . \end{aligned}$$

The temporal dynamics of $\eta (v,t)$ is given by the following evolution equation

5 $$\begin{aligned} \frac{\mathrm{d}\eta (v,t)}{\mathrm{d}t}=\phi (\eta (v,t),v,t), \end{aligned}$$

where $\phi (\eta (v,t),v,t)$ is the density operator of *F* in (2). Equations (3) and (4) introduced above for the measures can be rewritten in a similar form for the dynamics of densities. Several insightful examples of dynamics based on (5) are provided in Sect. 3.

#### Theorem 1

Element v is better than element w ($v\succ w$), if there exist neighbourhoods of these elements (*O*(*v*) of *v* and *O*(*w*) of *w*) such that the ratio of densities (6) tends to zero uniformly in these neighbourhoods $(\forall v'\in O(v)$ and $\forall w' \in O(w))$, i.e.

6 $$\begin{aligned} \frac{\eta (w',t)}{\eta (v',t)} \xrightarrow [\mathrm{uniformly}]{}0, t\rightarrow \infty . \end{aligned}$$

The proof of this theorem is given in ‘Appendix A’.

The importance of the assumption about uniform convergence in this theorem is crucial, as can be seen from the following example.

#### Example 2

Consider a self-replicating system where elements are described by a scalar parameter $v\in [0,1]$. For the sake of simplicity, we consider the measure density $\eta$ to be the population density $\rho$. Let the dynamics of the density $\rho (v,t)$ be described by the following equation which is of a logistic type

$$\begin{aligned} \frac{\mathrm{d} \rho (v,t)}{\mathrm{d}t}=k(v,t)\rho -\rho \int _{0}^{1}k(v,t)\rho \mathrm{d}v, \end{aligned}$$

where the reproduction coefficient *k* is time-dependent and given by $k=vt\exp (-vt)$; for simplicity consider the initial density $\rho (v,0)=1$.

Solving the above equation gives (see ‘Appendix B’)

$$\begin{aligned} \rho (v,t)=\frac{g(v,t)}{\int _{0}^{1}g(v,t)\mathrm{d}v}, \quad \text {where} \quad g(v,t)=\exp \big ( -t\exp (-vt)+(1-\exp (-vt))/v \big ). \end{aligned}$$

One can prove that $g(v,t)\rightarrow \exp (1/v)$, $v>0$ and $g(0,t)=1$. Moreover, the density tends to zero for all *v*, i.e. $\rho (v,t) \rightarrow 0$ for $t \rightarrow \infty$. On the other hand, for the measure of the interval [0, 1] we have

$$\begin{aligned} \mu (t)([0,1])=\int _{0}^{1}\rho (v,t)\mathrm{d}v=\frac{{\int _{0}^{1}g(v,t)\mathrm{d}v}}{\int _{0}^{1}g(v,t)\mathrm{d}v} \equiv 1. \end{aligned}$$

From this example, it follows that even if the density of the measure tends to zero for all elements of the set *A*, this does not signify that its measure $\mu (A)$ also vanishes at large times.

#### Remark

We should stress here that one should not confuse the density $\eta (v,t)$ with the ‘true’ population density: $\eta (v,t)$ should be considered as a particular characteristic (which is a function of the population density) for which if it tends to zero, the underlying population density also tends to zero. In this particular case, we have $\eta (v,t)\equiv \rho (v,t)$, where $\rho (v,t)$ is the ‘true’ population density (e.g. see Example 2).

### Constructing Evolutionary Fitness

The definition of evolutionary fitness stated in the previous section is axiomatic, in the sense that it does not provide a procedure for finding *J*(*v*). In this section as well as in Sect. 3, we demonstrate several ways of constructing evolutionary fitness using the underlying density equations.

We start with a construction of fitness using the long-term time average of the generalised reproduction coefficient (assuming that the average value exists, which might not be the case in some models) given by

7 $$\begin{aligned} \lim _{T\rightarrow \infty } \frac{1}{T}\int _{0}^{T} \frac{\eta '_t(v,t)}{\eta (v,t)}\mathrm{d}t = \Bigg \langle \frac{\eta '_t(v,t)}{\eta (v,t)} \Bigg \rangle \equiv J_1(v) \end{aligned}$$

Evolutionary fitness can be constructed using the following theorem.

#### Theorem 2

Assume the existence of a long-term time average (7) of the generalised reproduction coefficient for all elements *v* with nonzero initial density $\eta (v,0)$. Moreover, assume that for any point *v* there is a neighbourhood *O*(*v*), where convergence in limit (7) is uniform on *O*(*v*). In this case, from $J_1(v)> J_1(w)$ it follows that $v\succ w$. Thus, we can consider the long-term time average $J_1(v)$ as evolutionary fitness.

The proof of the theorem is given in ‘Appendix C’.

#### Corollary 2.1

If the conditions of Theorem 2 hold, $J_1(v)$ is a continuous functional and $v^*$ is a point of the global maximum of $J_1$ defined by (7), we then have $J_1(v^*)=0$.

The proof of Corollary 2.1 is given in ‘Appendix C’. Biologically, this signifies that the maximal average per capita growth rate should be zero.

#### Remark

In the proof of Theorem 2 (see ‘Appendix C’), it is shown that the fitness functional $J_1$ can be expressed as $J_1(v)=\lim _{T\rightarrow \infty }(\ln (\eta (T,v))/T)$.

#### Remark

The uniform convergence to the time average required in Theorem 2 is of crucial importance. Consider the equation introduced in Example 2. The formal computation of fitness in this case gives

$$\begin{aligned} J(v)=\Big \langle k(v,t) \Big \rangle -\Big \langle \int _{0}^{1}k(v,t)\eta \mathrm{d}v\Big \rangle =1-1=0. \end{aligned}$$

However, we cannot consider this function as evolutionary fitness since the convergence to the average per capita growth rate is not uniform on the interval [0, 1].

#### Remark

The function of evolutionary fitness depends on what we determine as the density $\eta$ of measures, which can be different from a biological definition, i.e. the population density. For example, it can be a certain power of the population density: $\eta =\rho ^{R(v)}$, where $\rho$ is the biological population density and $R(v)>0$ is a certain function(al) of *v*. The choice of the formulation of $\eta$ may depend on the particular biological study case.

Computing the time average per capita growth rate (7) is not the only possible way of finding evolutionary fitness. In particular, one can prove that a certain combination of model parameters can be considered as a fitness if it preserves the ranking order given by Definition 2. Interestingly, in some cases it is possible to construct an evolutionary fitness which does not depend on the initial conditions. Consider the following insightful example.

#### Example 3

Let the dynamics of the population density $\rho (v,t)$ be described by the following equation of logistic type

$$\begin{aligned} \frac{\mathrm{d} \rho (v,t)}{\mathrm{d}t}=k(v)\rho -r(v)\rho \int _{0}^{1}\rho \mathrm{d}v, \end{aligned}$$

where $v\in [0,1]$; *k*(*v*) and *r*(*v*) are positive functions of *v*.

We make the following change of variables $\eta (v,t)=\rho (v,t)^{1/r(v)}$. For the new variable $\eta$ (which can be considered as the generalised density of the measure), we obtain the following equation

$$\begin{aligned} \frac{\mathrm{d} \eta (v,t)}{\mathrm{d}t}=\frac{k(v)}{r(v)}\eta -\eta \int _{0}^{1}\eta (w)^{r(w)}\mathrm{d}w. \end{aligned}$$

For evolutionary fitness based on the long-term growth rate (7), we have

$$\begin{aligned} J_1(v)=\frac{k(v)}{r(v)}-\Big \langle \int _{0}^{1}\eta (w)^{r(w)} \mathrm{d}w \Big \rangle . \end{aligned}$$

Since the integral (second) term in the above expression is constant, from $k(v)/r(v)>k(w)/r(w)$ it follows (Theorem 2) that

8 $$\begin{aligned} \lim _{t\rightarrow \infty }\frac{\eta (w,t)}{\eta (v,t)}=\lim _{t\rightarrow \infty }\frac{\rho (w,t)^{1/r(v)}}{\rho (v,t)^{1/r(w)}}=0. \end{aligned}$$

As such, we can consider $\eta (v,t)=\rho (v,t)^{1/r(v)}$ as characteristics of the presence of a strategy within the population, i.e. as the generalised population density. This is possible since $\eta (w,t)\rightarrow 0$ will automatically indicate that $\rho (w,t)\rightarrow 0$. Thus, as evolutionary fitness in the model, we can take the ratio *k*(*v*) / *r*(*v*), which is independent of the initial conditions. Using $J=k(v)/r(v)$ can be more practical as compared to $J_1$ based on the long-term per capita growth rate (7).

#### Remark

The definition of fitness in Sect. 2.1 based on ranking order and its mathematical representation via the generalised reproduction coefficient (7) are extensions of the Darwinian idea of ‘Survival of the Fittest’. This, however, contains some seeds of tautology due to a deterministic interpretation of Darwin’s evolutionary theory: the fittest strategies will be those selected by long-term evolution, and the strategies selected by evolution will be the fittest ones. This is an a posteriori interpretation of fitness (also see Sect. 4). Establishing a rigorous definition of fitness, however, is a vital step between connecting the a posteriori and a priori concepts of fitness. The next section gives examples of a priori implementation of fitness which provides tools to create links between measurable/observable characteristics of organisms such as life traits or behavioural patterns (described by model coefficients) and long-term evolutionary outcomes, thus predicting which combination of biological parameters (under some constraints) should produce a successful strategy. We should stress that obtaining a mathematical expression for evolutionary fitness as a function(al) of model coefficients can be a difficult task. We show below that for some classes of models with age structuring one can derive evolutionary fitness and predict the evolution outcome. Moreover, some numerical procedures have recently been proposed for finding evolutionary fitness for a generic population model (see Sect. 4 for more details).

## Revealing Fitness in Some Age-Structured Models

Here we demonstrate how evolutionary fitness can be derived for several generic population models with age structure.

### Fitness in a Population Model with Discrete Stages

Consider the following model. Let $z(v,t)=(z_1(v,t),\ldots , z_n(v,t))$ be the vector of variables of the model corresponding to element *v*, an inherited behavioural strategy. Relations between the variables *z* have the form of the following differential equation

9 $$\begin{aligned} z'_t(v)=L(v)z(v)-R(v)f(z,t)z(v). \end{aligned}$$

Here *L*(*v*) is a matrix $[n\times n]$ with components $q_{ij}(v)$ independent on *t*, *R*(*v*) is a coefficient independent on *t*, and *f*(*z*, *t*) is a functional independent on *v*, for instance

$$\begin{aligned} f(z,t)=\int _{V} \sum _{i=1}^{n} z_i(v,t)\mu ^{*}(\mathrm{d}v). \end{aligned}$$

By introducing the normalising change of variables

$$\begin{aligned} \xi _i(v,t)= \frac{z_i(v,t)}{\sum _{i=0}^{n} z_i(v,t)}, \qquad \sum _{i=0}^{n} \xi _i(v,t)=1, \end{aligned}$$

we arrive at the following system for the frequencies $\xi (v,t)=(\xi _1(v,t),\ldots ,\xi _n(v,t)$

10 $$\begin{aligned} \frac{\mathrm{d}\xi (v,t)}{\mathrm{d}t}=L(v)\xi (v,t)-\xi (v,t)F(v,t), \end{aligned}$$

where $F(v,t)=\sum _{i=1}^{n} \sum _{j=1}^{n} q_{ij} \xi _j(v,t)$. The equation for the total population density $Z(v,t)=\sum _{i=0}^{n} z_i(v,t)$ is given by

11 $$\begin{aligned} \frac{\mathrm{d}Z(v,t)}{\mathrm{d}t}=\Big (F(v,t)-R(v)f(t)\Big )Z(v,t). \end{aligned}$$

Consider the following linear system

12 $$\begin{aligned} \frac{\mathrm{d}\zeta (v,t)}{\mathrm{d}t}=L(v)\zeta (v,t), \end{aligned}$$

where $\zeta (v,t)$ is a vector $(\zeta _1(v,t),\ldots ,\zeta _n(v,t))$. The solutions of (10) and (12) are related to each other via (see ‘Appendix D’ for detail)

13 $$\begin{aligned} \xi _i(v,t)= \frac{\zeta _i(v,t)}{\sum _{i=0}^{n} \zeta _i(v,t)}. \end{aligned}$$

We suggest, for simplicity, that the matrix *L* has only single eigenvalues $\lambda _i$ and, by consequence, has *n* linearly independent eigenvectors $e_i(v)$. Without loss of generality, we can always assume that the sum of components of each eigenvector is 1. We can sort the eigenvalues in descending order of their respective real values. The general solution of (12) with constants $c_i$ depending on initial condition reads as

14 $$\begin{aligned} \zeta (v,t)= \sum _{i=0}^{n} c_i e_i(v) \exp (\lambda _i(v,t)). \end{aligned}$$

We return to the variables $\xi _i(v,t)$ to obtain

15 $$\begin{aligned} \xi (v,t)= \frac{\sum _{i=0}^{n} c_i e_i(v) \exp (\lambda _i(v,t))}{\sum _{i=0}^{n} c_i \exp (\lambda _i(v,t))}. \end{aligned}$$

Let us assume that the initial condition is such that $c_1>0$ and $\lambda _1$ is a real number. In this case, for large times the solution of (10) will be dominated by the first eigenvector. This will be the asymptotic state of the model. The stationary state of (10) will be given by

16 $$\begin{aligned} 0=L(v)\xi (v)-\xi (v)F(v), \end{aligned}$$

One can easily see that the equilibrium state of (10) is the eigenvector of (12), and the value of *F* at equilibrium is equal to its eigenvalue, thus for large times $F \rightarrow \lambda _1$. Moreover, due to the continuous dependence of the model coefficients on *v*, we have $F \rightarrow \lambda _1$ uniformly. One can demonstrate in this case that the temporal average of *F* is $\lambda _1$. To characterise the presence of strategy *v* within the population, we will consider the quantity $\eta (v)=Z^{1/R(v)}(v,t)$, which satisfies all assumptions of the measure required in Sect. 2.1. The dynamics of $\eta (v)$ are described by the following equation

17 $$\begin{aligned} \frac{\mathrm{d}\eta (v,t)}{\mathrm{d}t}=F(v,t)\eta (v,t)/R(v)-f(z,t)\eta (v,t). \end{aligned}$$

According to Theorem 1, the fitness is given by

18 $$\begin{aligned} J=\Big \langle \frac{F(v,t)}{R(v)}\Big \rangle -\langle f \rangle =\frac{\lambda _1(v)}{R(v)}-\langle f \rangle , \end{aligned}$$

which is equivalent to

19 $$\begin{aligned} J_1=\frac{\lambda _1(v)}{R(v)}. \end{aligned}$$

A clear advantage of using $J_1$ rather than *J* is that the former does not involve $\langle f \rangle$, which would need to be computed. Moreover, $J_1$ does not depend on initial conditions which simplifies our computation of its optimal value.

For example, consider a population with *n* developmental stages. We denote $z_i(v)$ to be the population density of stage *i* individuals using strategy *v*. We assume that all stages (except the newly produced individuals with $i=1$) can contribute to the production of newborns, which is described by the reproduction coefficient $b_i(v)$: without losing any generality we can consider that some $b_i(v)$ are zero. The individuals are subject to natural mortality (in the absence of competition and predation) at a rate of $a_i(v)$. The model equations for $z_i(v)$ read

20 $$\begin{aligned} \frac{\mathrm{d}z_1(v,t)}{\mathrm{d}t}= & {} \sum _{i=2}^{n} b_{i}(v)z_{i}(v,t)- p_1(v)z_1(v,t)\nonumber \\&-\,a_1(v)z_1(v,t)-R(v)y(t)z_1(v,t), \end{aligned}$$

21 $$\begin{aligned} \frac{\mathrm{d}z_i(v,t)}{\mathrm{d}t}= & {} p_{i-1}(v)z_{i-1}(v,t)-p_i(v)z_i(v,t)-a_i(v)z_i(v,t) \nonumber \\&-\,R(v)y(t)z_i(v,t), 2 \le i\le n-1 \end{aligned}$$

22 $$\begin{aligned} \frac{\mathrm{d}z_n(v,t)}{\mathrm{d}t}= & {} p_{n-1}(v)z_{n-1}(v,t)-a_n(v)z_n(v,t)-R(v)y(t)z_n(v,t), \end{aligned}$$

where $p_i(v)$ describes the transition rate from stage *i* into $i+1$ due to ageing; *y*(*t*) is some growth limitation factor determining extra mortality within the population (due to predation, interspecific competition, etc); *R*(*v*) is the coefficient describing the strength of the influence of the limitation factor *y* on the individuals using strategy *v*. We assume that all coefficients in (20)–(22) are continuous functionals of the strategy *v*.

This system is the partial case of (9), where $f=y$, $q_{11}=-p_1-a_1$, $q_{1i}=b_i$, $q_{ii-1}=p_{i-1}$, $q_{ii}=-p_i-a_i$, $2 \le i\le n-1$, $p_n=0$. The above results provide the function of generalised fitness (19) for the given model.

### Fitness in a Generic Population Model with Delay

Consider the following model. Let $z(v,t)=(z_1(v,t),\ldots , z_n(v,t))$ be the vector of variables of the model, corresponding to element *v*, an inherited behavioural strategy. Relations between the variables *z* have the form of the following differential equation

23 $$\begin{aligned} \frac{\mathrm{d}z_l(v,t)}{\mathrm{d}t}= & {} \sum _{j=1}^{n} \sum _{i=1}^{m}q_{lji}(v)z_j (v,t-\tau _{i}^{*})\exp (-R(v)\int _{t-\tau _{i}^{*}}^{t}f(z,t)\mathrm{d}t)\nonumber \\&+\sum _{j=1}^{n}r_{lj}(v)z_j(v,t)-R(v)z_l(v,t)f(z,t), 1\le l\le n. \end{aligned}$$

where $q_{lji}(v)$, $r_{lj}(v)$, *R*(*v*) are continuous functionals over *V*; *f*(*z*, *t*) is a continuous functional.

In the particular case of $n=1$, we can consider Eq. (23) as the equation for the density of the measure described by Eq. (4).

The change of variables

24 $$\begin{aligned} z(v,t)\exp (R(v)\int _{0}^{t}f(z,t)\mathrm{d}t)= \zeta (v,t), \end{aligned}$$

where $\zeta$ is the vector $(\zeta _1(t),\ldots ,\zeta _n(t))$, transforms this equation into the following simplified version

25 $$\begin{aligned} \frac{\mathrm{d} \zeta _l(t)}{\mathrm{d}t}=\sum _{j=1}^{n} \sum _{i=1}^{m}q_{lji}(v)\zeta _j (t-\tau _{i}^{*})+\sum _{j=1}^{n}r_{lj}(v)\zeta _j(t), 1\le l\le n. \end{aligned}$$

The proof is provided in ‘Appendix E’.

We now seek the solution $\zeta =(\zeta _1(t),\ldots ,\zeta _n(t))$ as a sum of $e_i\exp (\lambda _i t)$, where $e_i =(e_{i1},e_{i2},\ldots ,e_{in})$ is a constant vector (eigenvector). The system of characteristic equations for the eigenvalues $\lambda$ reads as

26 $$\begin{aligned} \det (H(\lambda )-\lambda E)=0, \end{aligned}$$

where *E* is the identity matrix; *H* is the matrix $[n\times n]$ with components $h_{lj}(\lambda ,v)=\sum _{i=1}^{m}q_{lji}(v)\exp (-\lambda \tau _{i}^{*})+r_{lj}(v)$, $e_i$ are the corresponding nontrivial solutions of the following system

$$\begin{aligned} \lambda =\sum _{j=1}^{n}\sum _{i=1}^{m} e_{ij} q_{lji}(v)\exp (-\lambda \tau _{i}^{*})+\sum _{j=1}^{n} e_{ij} r_{lj}(v), 1\le l\le n. \end{aligned}$$

The general solution $\zeta$ with constants $c_i$ depending on initial condition reads as

27 $$\begin{aligned} \zeta (v,t)= \sum _{i=0}^{\infty } c_i e_i(v) \exp (\lambda _i(v,t)). \end{aligned}$$

We rank the solutions $\lambda$ of the characteristic equation in descending values of their real parts ($\lambda _1$ having the largest real part) and assume that $c_1 \ne 0$. We can characterise the presence of strategy *v* in the population by $\eta (v,t)=(\sum _{j=1}^{n}z_j(v,t))^{1/R(v)}$ since it satisfies all assumptions stated in Sect. 2. We further consider the following limit

$$\begin{aligned} \lim _{t\rightarrow \infty } \frac{\eta (w,t)}{\eta (v,t)}=\lim _{t\rightarrow \infty } \frac{\Big (\sum _{i}c_i(w)\exp (\lambda _i(w)t)\Big )^{1/R(w)}}{\Big (\sum _{i}c_i(v)\exp (\lambda _i(v)t)\Big )^{1/R(v)}}. \end{aligned}$$

One can see that the above limit will be zero in the case where $\max _{i}\mathfrak {R}(\lambda _i(v))/R(v)>\max _{i}\mathfrak {R}(\lambda _i(w))/R(w)$, here $\mathfrak {R}(\lambda _i(v))$ is the real part of $\lambda _i(v)$. Since all coefficients of the characteristic equation are continuous functions of *v*, its solution remains a continuous function. One can easily show that the above limit is uniform in some small neighbourhoods of *u* and *v*. Therefore, we can consider the function $J(v)=\max _{i}\mathfrak {R}(\lambda _i(v))/R(v)$ as evolutionary fitness. This fitness does not depend on the initial conditions as far as they are chosen in a way that the coefficient $c_1$ is nonzero.

#### Example 4

Consider a single population model with structuring described by a Foerster’s type equation (Botsford et al. 1994; Cushing 1998). The population is characterised by its density $u(v, t, \tau )$ at the moment of time *t* with age $\tau$ and behavioural strategy *v*.

28 $$\begin{aligned} \frac{\partial u(v,\tau , t)}{\partial t} +\frac{\partial u(v,\tau , t)}{\partial \tau } = -A(v, \tau )u(v,\tau , t)- R(v)y(t) u(v,\tau , t), \end{aligned}$$

where $A(v, \tau )$ is the linear (natural) mortality rate; the second mortality term has the same meaning as in (20)–(22). The production of offspring is due to the reproduction of the whole cohort of adults which is given by

29 $$\begin{aligned} u(v,0, t)=\int _{\tau _1}^{+\infty } b(v,\tau ) u(v,\tau , t)\mathrm{d}\tau , \end{aligned}$$

where $b(v,\tau )$ is the reproduction coefficient and $\tau _1$ is the minimum reproductive age.

We further split the entire population into *n* age groups each of which has a particular strategy; the different stages are described by the following life trait parameters

$$\begin{aligned} A(v,\tau )= & {} {\left\{ \begin{array}{ll} a_1(v),&{} 0 \le \tau< \tau _1,\\ a_i(v), &{} \tau _{i-1} \le \tau< \tau _{i},\quad 2 \le i\le n-1 \\ a_n(v), &{} \tau _{n-1} \le \tau< +\infty , \\ \end{array}\right. }\\ b(v, \tau )= & {} {\left\{ \begin{array}{ll} b_i(v), &{} \tau _{i-1} \le \tau< \tau _{i},\quad 2 \le i\le n-1 \\ b_n(v), &{} \tau _{n-1} \le \tau < +\infty . \\ \end{array}\right. } \end{aligned}$$

We further introduce the following integral quantities for the total population densities for stage *i*.

30 $$\begin{aligned} S_i(v,t)=\int _{\tau _{i-1}}^{\tau _i} u(v,\tau , t)\mathrm{d}\tau , \quad 1 \le i\le n, \quad \tau _0=0, \quad \tau _{n}=+\infty . \end{aligned}$$

We recast Eq. (28) into the equation for $S_i(t)$

31 $$\begin{aligned} \frac{\mathrm{d} S_i(v, t)}{\mathrm{d} t} +u(v,t,\tau _{i}) -u(v,t,\tau _{i-1}) = -a_i(v)S_i(v, t)- R(v)y(t) S_i(v, t), \end{aligned}$$

where $$1 \le i\le n$$ and $$u(\tau _{n})=0$$.

Integration of (28) from $\tau _{i-1}$ to $\tau _{i}$ gives

32 $$\begin{aligned} u(v, t, \tau ) =u(v,t-(\tau -\tau _{i-1}), \tau _{i-1})\exp \Big (-a_{i}(v)(\tau -\tau _{i-1})- R(v)\int _{t-(\tau -\tau _{i-1})}^{t} y(t)\mathrm{d}t\Big ), \end{aligned}$$

where $\tau _{i-1}<\tau < \tau _{i}$, $1 \le i\le n$ with $\tau _0=0$. The boundary condition for *u*(*t*, 0, *v*) is given by

33 $$\begin{aligned} u(v,t,0)=\sum _{i=2}^{n}b_i(v)S_i(v,t). \end{aligned}$$

We substitute (32) into (31) to obtain

34 $$\begin{aligned}&\frac{\mathrm{d} S_i(v, t)}{\mathrm{d} t} \nonumber \\&\quad =\sum _{k=2}^{n}b_k(v)S_k(v,t-\tau _{i-1})\exp \Big (-\sum _{k=1}^{i-1}a_k(v)(\tau _{k}-\tau _{k-1})-R(v)\int _{t-\tau _{i-1}}^{t}y(t)\mathrm{d}t\Big )\nonumber \\&\qquad -\sum _{k=2}^{n}b_k(v)S_k(v,t-\tau _{i})\exp \Big (-\sum _{k=1}^{i}a_k(v)(\tau _{k}-\tau _{k-1})-R(v)\int _{t-\tau _{i}}^{t}y(t)\mathrm{d}t\Big )\nonumber \\&\qquad -a_i(v)S_i(v, t)- R(v)y(t) S_i(v, t), \end{aligned}$$

where $1 \le i\le n$. Note that for the last stage $i=n$, the second term in (34) should be removed.

The given system is a particular case of above model (23), where $f=y$, $m=n-1$, $q_{ljl-1}=b_j(v)\exp \Big (-\sum _{k=1}^{l-1}a_k(v)(\tau _{k}-\tau _{k-1})\Big )$, $q_{ljl}=-b_j(v)\exp \Big (-\sum _{k=1}^{l}a_k(v)(\tau _{k}-\tau _{k-1})\Big )$, $r_{lj}=-a_l(v)$, other coefficients are equal to zero.

Therefore, the generalised fitness for the population is given by

$$\begin{aligned} J=\frac{\max _{i}\mathfrak {R}(\lambda _i(v))}{R(v)}, \end{aligned}$$

where $\lambda _i$ is the solution of the characteristic equation (26). Note that this fitness functional does not depend on the initial conditions.

## Discussion and Conclusions

Fitness is one of the most influential concepts in evolutionary modelling following the seminal idea of Sewall Wright concerning a hypothetical adaptive fitness landscape (Wright 1932, 1988). The metaphor of ‘climbing uphill’ to reach a local peak is compelling and easily graspable, which has probably helped to make the concept of fitness so popular in the literature. However, the initial idea by Wright has also met with criticism from many different backgrounds. A major point of criticism centres on the fact that the shape of the fitness landscape should not be stationary, but instead should constantly change in the course of evolution due to a permanent dynamical feedback between the strategies of individuals and the environment (Nowak and Sigmund 2004). To rectify this situation, more advanced approaches have been proposed. In particular, adaptive dynamics introduces the well-known concept of invasion fitness, which provides the condition for the invasion of a rare mutant in an environment set by a resident type. Since the resident type is changed via consecutive replacements of successful mutants, the overall fitness landscape should be constantly being varied until we arrive at an evolutionary endpoint and the evolution-environment feedback loop is captured (Geritz et al. 1997, 1998; Parvinen et al. 2006).

Our revised concept of fitness is actually an extension of the idea of Wright in that we still assume that the evolutionary outcome should maximise some fitness. However, we also take into account the dynamical feedback between the population and the environment. Our idea of fitness is closely linked to the concept of the ranking order of strategies, given by the long-term limit of the ratio of their densities (6). The ranking order and thus the definition of evolutionary fitness may both depend on what we understand by the density of measures $\eta (v)$, which should not necessarily be some ecological density $\rho (v)$ such as the population size or biomass per volume, but could also be a function of such a density, possibly depending on the strategy *v* itself. For example, we might choose $\eta (v)=\rho (v)^{R(v)}$, where *R*(*v*) is an arbitrary continuous positive functional on *V*, in which case changing *R*(*v*) may give us a different ranking order. The choice of measure density $\eta (v)$ can be based on the underlying equations for the population density $\rho (v)$ (see Example 3 in Sect. 2 and Sects. 3.1, 3.2), but this is not necessary. The main requirement is that using some other density $\eta$ should not change the evolutionary outcome of selection, i.e. the maximum fitness strategies remain the same even if the ordering of less fit strategies changes. We should also emphasise that the ranking order (6) might strongly depend on initial conditions, i.e. the initial presence/absence of other strategies.

The framework for modelling selection processes proposed here is based on exploring the long-term dynamics of measures in the space of strategies, as suggested in some earlier works Cressman and Hofbauer (2005), Gorban (2007). This approach is generic, so it can be equally applied to modelling selection in chemistry, sociology, turbulence theory and economics. Although realistic populations are discrete, in many cases, we have the situation where a large number of subpopulations (genotypes) with close traits simultaneously compete with each other, so that we can use a continuum approximation. This is relevant to biological reality, where in studies we usually compare competitive efficiency of some traits against each other: we technically deal with subpopulations with life traits located within some continuum ranges. However, we show here that we should implement the density-based modelling framework with care and compare particular strategies with each other only under some mathematical restrictions (uniform convergence). In this case, we actually do not only compare pure strategies *w* and *v* per se but also their surroundings; thus, we still appeal to the overall measure-based framework.

The proposed evolutionary fitness concept using ranking order of strategies is actually an extension of Darwin’s ‘Survival of the Fittest’ idea. This implies, in particular, that the fittest strategies are those which will eventually survive after a very long time, and so their fitness *J* will be maximal. We should say, however, that only postulating such a definition is in a certain sense tautological as it follows from the deterministic interpretation of Darwinian evolutionary theory. Indeed, the inherited unit(s) selected by long-term evolution will be the fittest one(s) and, inversely, the fittest inherited unit(s) should be those which will eventually survive (Mills and Beatty 1979). In this case, the idea of fitness becomes an a posteriori rather than an a priori concept. However, we should still stress that the formulated definition allows us to analytically and/or computationally more easily derive which strategies are the fittest. Moreover, creating a rigorous definition is a necessary step to sort out the above tautological situation.

In Sect. 3, we show a possible way of how the above mentioned tautological aspects can be resolved and then used as an a priori framework. The main idea is that for the given environment (deterministic or stochastic) we can establish links between measurable characteristics of organisms such as life traits or behavioural patterns and long-term evolutionary outcomes. In other words, our a priori knowledge of life traits or behavioural strategies should allow us to predict long-term evolutionary outcome provided we have enough information about intra-specific and interspecies interactions as well as the environment (mathematically, such information is fixed in terms of algebraic or/and differential constraints representing the model). For example, the strategy maximising the ratio between the reproduction rate and mortality is shown to be eventually dominant in a simple system under variable predation, provided the model itself is correct (Morozov and Kuzenkov 2016). In more complex models, the expression for fitness will be obviously more complicated (Sect. 3). Therefore, the major goal is to provide a mathematical function(al) expressing the link between observable life traits or behaviours (i.e. a priori information) and the long-term evolution success. Here the definition of fitness serves as major tool for constructing such a link. Finally, we should also stress that we explore evolution and selection in the absence of rare or singular catastrophic events (e.g. a single earthquake, forest fire, atomic explosion) which might results in some paradoxical misinterpretation of fitness [see Examples in Mills and Beatty (1979)].

The fitness *J* introduced here allows us to formulate the variational principle of modelling evolutionary dynamics: the outcome of long-term selection should correspond to the maximum of the fitness functional across the strategies initially present. Ideas of optimisation in evolution have been suggested in earlier works, for example in the adaptive dynamics framework, where it was found that evolution will optimise the invasion fitness of a mutant introduced in the resident population in the case where the environment affects fitness in an effectively monotone one-dimensional manner (Metz et al. 1992, 2008). However, generic classes of models for which this property holds are still poorly understood (Gyllenberg et al. 2011). For example, it has been shown that the optimisation principle (i.e. the existence of a certain function(al) whose maximisation would provide the evolutionary attractor) should require the absence of so-called rock–scissors–paper cycles of invasion of mutants into the environment set by a resident (Gyllenberg and Service 2011). We should stress that the meaning of evolutionary optimisation in the current paper is somewhat different to the one considered in the adaptive dynamics framework (Gyllenberg and Service 2011; Metz et al. 1992, 2008), since it does not implement the invasion-replacement paradigm of adaptive dynamics. In particular, in our case, the strategy which maximises the fitness *J* may depend on the initial conditions, as in Example 1.

Here we have considered selection processes in deterministic systems, but similar definitions can be formulated in systems with environmental or demographic noise, where we should consider dynamics of expected measures. In particular, the introduced ranking order can then be defined as the ratio of expected measures. However, adding noise would make the derivation of fitness function(al)s more complicated. For example, we would need to include information about the covariances of species growth rates: even for simple density-independent dynamics, computing the geometric mean of the growth rate (known as ‘Malthusian fitness’) will not provide a correct fitness function (Lande 2007). Considering more complicated dynamics via a generalised logistic equation with noise would make the evolutionary outcome even more complicated: one would need to maximise the expected value of the density-dependent component of the growth rate (Lande et al. 2009). However, even this maximisation principle still does not seem to be generic enough to cover other more complicated models. Another issue related to stochasticity is that our current Definitions in Sect. 2 do not allow for the possibility of stochastic extinction of a subpopulation when its measure (e.g. the population size) drops to a very low value. However, we can incorporate this as well by setting a certain threshold $\epsilon$ for the measures/densities and assuming that reaching this threshold would signify the extinction of species. Note that in this case, the overall axiomatic approach towards modelling selection will be similar to the current one.

It would be interesting to compare predictions of long-term selection using the current approach with some other well established approaches, such as adaptive dynamics, for example. Strictly speaking, a rigorous direct comparison between our approach and that of adaptive dynamics is hardly possible at all, since in the current settings we assume the absence of ongoing mutations. Also, a fair comparison would require us to have an ergodic environment, as is required for adaptive dynamics (Metz et al. 1992, 2008), so adaptive dynamics would not be applicable to Example 2. However, one can suggest that we already include some mutations in our settings when we consider arbitrary nonzero initial strategies in the space of strategies so that various ‘mutations’ are already present in the system at the start and the fittest mutation will grow and outcompete the other strategies. As such, one can consider the scenario where initial nonzero densities (‘mutations’) are distributed in the vicinity of the strategy $v^*$ maximising the fitness *J* given by Definition 3 (applied to the scenario where initial nonzero densities are located in a small vicinity of $v^*$). In principle, we can also consider a polymorphic case of several strategies which maximise the fitness: $J(v^*)=J(u^*)=\cdots =J(w^*)$ and consider initial mutations in the vicinity of each of them. To our understanding, it seems that $v^*$ will also be an evolutionary attractor in the sense of adaptive dynamics (although a strict mathematical proof of this still needs to be done). Indeed, adaptive dynamics requires that an evolutionary attractor should be convergence stable, non-invasible and, in the case of protected polymorphism, mutation strategies closer to $v^*$ should be able to invade (Geritz et al. 1997, 1998). Our definition of fitness covers all of these properties by requiring uniform convergence in (6). For example, uniform convergence in the vicinity of $v^*$ in our definition of fitness rules out the possibility of a ‘Garden of Eden’ situation, in which the best strategy $v^*$ outcompetes all other strategies, but taking out this strategy from the initial nonzero set would drastically change the evolutionary outcome (Broom and Rychtar 2013). The question about the possibility of achieving the global maximum of *J* via small mutations in adaptive dynamics remains an open one since the evolutionary trajectory can get stuck at a local attractor.

In this paper, using a specific change of variables we derived evolutionary fitness for some classes of population models (Sect. 3). The question of how to reveal a population fitness for some more complicated models remains. The development of efficient analytical methods to deal with other classes of population models will be the priority for our next work, but the outcome of selection in self-reproducing systems with high complexity can also be obtained via numerical methods which take into account the theoretical framework of this paper, in particular, the requirement of uniform convergence in the space of strategies. For example, in recent work Sandhu et al. (2017), it was shown how the best strategy $v^*$ can be found using a straightforward computational algorithm. The method can be used for both scalar and function-valued traits and can also be implemented in situations where we do not know the underlying dynamical equations. However, the proposed method is based on the assumption that the fitness does not depend on initial conditions, and so it should be modified to deal with more realistic situations.

## Appendix A

Let there exist neighbourhoods *O*(*v*) and *O*(*w*) of points *v* and *w* such that the ratio of densities $\eta (w',t)/\eta (v',t)$ tends to zero uniformly in *O*(*v*) and *O*(*w*). In other words, for any $\epsilon >0$ there should be $T>0$ such that for any $t>T$ for any $w'$ from *O*(*w*) and for any $v'$ from *O*(*v*) the following inequality holds

$$\begin{aligned} \frac{\eta (w',t)}{\eta (v',t)} <\epsilon . \end{aligned}$$

Moreover, any neighbourhoods of points *v* and *w* are $\mu$-nonzero, hence *O*(*v*) and *O*(*w*) are also $\mu$-nonzero. Let *A* be any $\mu$-nonzero subset from *O*(*w*). Then neighbourhoods *O*(*v*), *O*(*w*) and the subset *A* are also $\mu ^*$-nonzero due to the absolute continuity of the measure $\mu$ with respect to the measure $\mu ^*$. Thus, the measures $\mu ^*$ of *O*(*v*), *O*(*w*) and *A* will be given by

$$\begin{aligned} \mu ^*(O(w))=\int _{O(w)}\mu ^*(\mathrm{d}w')=\alpha>0 \qquad \text {and} \qquad \mu ^*(A)=\int _{A}\mu ^*(\mathrm{d}v')=\gamma >0. \end{aligned}$$

By integrating the ratio of densities in the neighbourhood *O*(*w*), we obtain

$$\begin{aligned} \int _{O(w)}\frac{\eta (w',t)}{\eta (v',t)}\mu ^*(\mathrm{d}w')= & {} \frac{1}{\eta (v',t)}\int _{O(w)}\eta (w',t)\mu ^*(\mathrm{d}w')\\= & {} \frac{1}{\eta (v',t)}\mu (t)(O(w))<\epsilon \alpha . \end{aligned}$$

In other words, we have

$$\begin{aligned} \frac{\eta (v',t)}{\mu (t)(O(w))}>\frac{1}{\epsilon \alpha }. \end{aligned}$$

By integrating the latter inequality in the set *A*, we obtain

$$\begin{aligned} \int _{A}\frac{\eta (v',t)}{\mu (t)(O(w))}\mu ^*(\mathrm{d}v')= & {} \frac{1}{\mu (t)(O(w))}\int _{A}\eta (v',t)\mu ^*(\mathrm{d}v')\\= & {} \frac{1}{\mu (t)(O(w))}\mu (t)(A)>\frac{\gamma }{\epsilon \alpha }. \end{aligned}$$

In other words, we have

$$\begin{aligned} \frac{\mu (t)(O(w))}{\mu (t)(A)}<\frac{\epsilon \alpha }{\gamma }. \end{aligned}$$

Therefore, the ratio of measures of the neighbourhood of point *w* and any subset *A* of the neighbourhood of point *v* becomes an arbitrarily small for big enough time *T* that means convergence of this ratio to zero. Therefore, any nontrivial subset of the neighbourhood of *v* is better than the neighbourhood of *w* and the neighbourhood of *v* is strong better than the neighbourhood of *w*. This finalises the proof of the theorem.

## Appendix B

Here we analytically find the solution in Example 2 (Sect. 2.2).

We consider the following auxiliary homogeneous equation

$$\begin{aligned} \frac{\mathrm{d}\sigma (v,t)}{\mathrm{d}t}=k(v,t) \sigma (v,t) \end{aligned}$$

with the same initial condition $\sigma (v,0)=1$.

One can see that the solutions of the above auxiliary homogeneous equation and the original model are connected via the following change of variables

$$\begin{aligned} \rho (v,t)=\frac{\sigma (v,t)}{\int _{0}^{1}\sigma (w,t)\mathrm{d}w}. \end{aligned}$$

We can easily integrate the homogeneous equation to obtain

$$\begin{aligned} \sigma (v,t)=\exp \Big (\int _{0}^{t}k(v,t)\mathrm{d}t\Big )=\exp \Big (-t\exp (-vt) +\frac{1}{v} - \frac{\exp (-vt)}{v} \Big ). \end{aligned}$$

For the solution of the original equation in terms of $\rho (v,t)$, we have

$$\begin{aligned} \rho (v,t)=\frac{\exp \Big (-t\exp (-vt) +\frac{1}{v}- \frac{\exp (-vt)}{v} \Big )}{\int _{0}^{1}\exp \Big (-t\exp (-vt) +\frac{1}{v} -\frac{\exp (-vt)}{v} \Big )\mathrm{d}w} . \end{aligned}$$

## Appendix C

Proof of Theorem 2.

Let $J(v)>J(w)$. Then there is a neighbourhood *O*(*v*) of the point *v* and a neighbourhood *O*(*w*) of the point *w* such that

$$\begin{aligned} \frac{1}{T}\int _{0}^{T} \frac{\eta '_t(v',t)}{\eta (v',t)}\mathrm{d}t \xrightarrow [T\rightarrow \infty ]{}J(v') \quad \text {and} \quad \frac{1}{T}\int _{0}^{T} \frac{\eta '_t(w',t)}{\eta (w',t)}\mathrm{d}t \xrightarrow [T\rightarrow \infty ]{}J(w') \end{aligned}$$

uniformly in *O*(*v*) and *O*(*w*), respectively. Moreover, for the same neighbourhoods we have $\inf _{O(v)}J(v')> \sup _{O(w)} J(w')$.

From computation of the long-term average growth rate, we have

$$\begin{aligned} J_1(v')=\lim _{T\rightarrow \infty }\frac{1}{T}\int _{0}^{T} \frac{\eta '_{t} (v',t)}{\eta (v',t)}\mathrm{d}t=\lim _{T\rightarrow \infty }\frac{1}{T}\ln (\eta (v',T)). \end{aligned}$$

Consider now the limit of the densities ratio in *O*(*v*) and *O*(*w*).

$$\begin{aligned} \lim _{T\rightarrow \infty }\frac{\eta (w',T)}{\eta (v',T)}= & {} \exp \Big (\lim _{T\rightarrow \infty } (\ln (\eta (w',T))- \ln (\eta (v',T)) \Big )\\= & {} \exp \Big (\lim _{T\rightarrow \infty } T\frac{(\ln (\eta (w',T))- \ln (\eta (v',T))}{T} \Big ) \\= & {} \lim _{T\rightarrow \infty }\exp \Big ( T (J_1(w')- J_1(v'))\Big )=0. \end{aligned}$$

The above limit is uniform in *O*(*v*) and *O*(*w*), since the convergence to the time average per capita growth rate (7) is uniform. This finalises the proof of the theorem.

We will now prove the corollary of Theorem 2 (Corollary 2.1).

Proof of Corollary. We assume that $J_1(v^*) \ne 0$. We firstly consider the case where $J_1(v^*) =\alpha >0$. Since $J_1(v)$ is continuous and there exists a neighbourhood $O(v^*)$ of $v^*$ such that $\inf _{v}J_1(v)> \alpha /2$ for *v* from $O(v^*)$ and convergence to the time average $J_1(v)$ is uniform for $v \in O(v^*)$. For the measures we have

$$\begin{aligned} \lim _{t\rightarrow \infty }\mu (t)(O(v^*))= & {} \lim _{t\rightarrow \infty }\int _{O(v^*)}\eta (v,t)\mu ^*(\mathrm{d}v)=\int _{O(v^*)}\lim _{t\rightarrow \infty }\eta (v,t)\mu ^*(\mathrm{d}v)\\= & {} \int _{O(v^*)}\exp \Big ( \lim _{t\rightarrow \infty }t\frac{\ln (\eta (v,t))}{t}\Big )\mu ^*(\mathrm{d}v)\\= & {} \int _{O(v^*)}\exp \Big (\lim _{t\rightarrow \infty }t J_1(v)\Big )\mu ^*(\mathrm{d}v) =+\infty . \end{aligned}$$

This contradicts the assumption on the boundedness of the measure of *V*.

Let us assume that $J_1(v^*) =\alpha <0$. Then for the measure of some neighbourhood *O*(*w*) we have

$$\begin{aligned} \lim _{t\rightarrow \infty }\mu (t)O(w)= & {} \lim _{t\rightarrow \infty }\int _{O(w)}\eta (w',t)\mu ^*(\mathrm{d}w')=\int _{O(w)}\lim _{t\rightarrow \infty }\eta (w',t)\mu ^*(\mathrm{d}w')\\= & {} \int _{O(w)}\exp \Big ( \lim _{t\rightarrow \infty }t\frac{\ln (\eta (w',t))}{t}\Big )\mu ^*(\mathrm{d}w')\\= & {} \int _{O(w)}\exp \Big (\lim _{t\rightarrow \infty }t J_1(w)\Big )\mu ^*(\mathrm{d}w)\\\le & {} \int _{O(w)}\exp \Big (\alpha \lim _{t\rightarrow \infty }t \Big )\mu ^*(\mathrm{d}w') =0. \end{aligned}$$

Since *V* is compact and a finite system of neighbourhoods $O(w_i)$ can cover *V*, for the measures we have

$$\begin{aligned} \lim _{t\rightarrow \infty }\mu (t)(V) \le \lim _{t\rightarrow \infty } \sum _{i=1}^{N} \mu (t)(O(w_i)) =0. \end{aligned}$$

This contradicts the assumption about the positivity of the measure of *V*. Therefore, we have $J(v^*)=0$.

## Appendix D

Let $\zeta$ be the solution of differential equation (12). We differentiate the expression of $\xi$ in (13) to obtain

$$\begin{aligned} \frac{\mathrm{d} \xi _l}{\mathrm{d}t}= & {} \frac{\zeta '_i}{\sum _{j=0}^{n} \zeta _j}-\frac{\zeta _i\sum _{j=0}^{n} \zeta '_j}{\left( \sum _{j=0}^{n} \zeta _j\right) ^2} \\= & {} \frac{\sum _{j=1}^{n} q_{ij} \zeta _j}{\sum _{j=0}^{n} \zeta _j}-\frac{\zeta _i\sum _{k=1}^{n} \sum _{j=1}^{n} q_{kj} \zeta _j}{\left( \sum _{j=0}^{n} \zeta _j\right) ^2} \\= & {} \sum _{j=1}^{n} q_{ij} \xi _j-\xi _i\sum _{k=1}^{n} \sum _{j=1}^{n} q_{kj} \xi _j=\sum _{j=1}^{n} q_{ij} \xi _j-\xi _iF. \end{aligned}$$

Therefore, $\xi$ satisfies Eq. (10).

## Appendix E

We differentiate the expression of $\zeta$ in the change of variable to obtain

$$\begin{aligned} \frac{\mathrm{d} \zeta _l(t)}{\mathrm{d}t}= & {} \frac{\mathrm{d}z_l(t)}{\mathrm{d}t}\exp \int _{0}^{t}R(v)f(z,t)\mathrm{d}t+z_l(t)R(v)f(z,t)\exp \int _{0}^{t}R(v)f(z,t)\mathrm{d}t\\= & {} \Bigg (\sum _{j=1}^{n} \sum _{i=1}^{m}q_{lji}(v)z_j (v,t-\tau _{i}^{*})\exp (-R(v)\int _{t-\tau _{i}^{*}}^{t}f(z,t)\mathrm{d}t\Bigg )\exp \int _{0}^{t}R(v)f(z,t)\mathrm{d}t \\&+\left( \sum _{j=1}^{n}r_{lj}(v)z_j(v,t)-R(v)z_l(v,t)f(z,t)\right) \exp \int _{0}^{t}R(v)f(z,t)\mathrm{d}t\\&+\,z_l(t)R(v)f(z,t)\exp \int _{0}^{t}R(v)f(z,t)\mathrm{d}t \\= & {} \sum _{j=1}^{n} \sum _{i=1}^{m}q_{lji}(v)z_j (v,t-\tau _{i}^{*})\exp (-R(v)\int _{0}^{t-\tau _{i}^{*}}f(z,t)\mathrm{d}t\\&+\left( \sum _{j=1}^{n}r_{lj}(v)z_j(v,t)\right) \exp \int _{0}^{t}R(v)f(z,t)\mathrm{d}t\\= & {} \sum _{j=1}^{n} \sum _{i=1}^{m}q_{lji}(v)\zeta _j (t-\tau _{i}^{*})+\sum _{j=1}^{n}r_{lj}(v)\zeta _j(t). \end{aligned}$$
